# Aspiration pneumonia with broncho-œsophageal fistula: a rare clinical image

**DOI:** 10.11604/pamj.2024.48.7.43032

**Published:** 2024-05-06

**Authors:** Mansi Deshmukh, Lajwanti Lalwani

**Affiliations:** 1 Department of Cardio Respiratory Physiotherapy, Ravi Nair Physiotherapy College, Datta Meghe Institute of Higher Education and Research, Sawangi (Meghe), Wardha, Maharashtra, India

**Keywords:** Broncho- œsophageal fistula, aspiration pneumonia, endoscopy

## Image in medicine

Broncho-œsophageal fistulas (BEF) are rarer than tracheœsophageal fistulas and can occur at birth or later in life. They may develop in adults due to various causes like malignancy, trauma, or infection. Diagnosis involves bronchoscopy, gastrointestinal endoscopy, or radiologic imaging. Management depends on symptom severity, fistula location, and patient health. Palliative options include metallic stents or surgical œsophageal bypass. Metallic stents have shown superiority. A 25-year-old female presented with fever, cough, vomiting, and weight loss. Imaging revealed consolidation with cavitation in the lower lobe communicating with the œsophagus, diagnosed as aspiration pneumonia. Endoscopy confirmed a suspected bronchœsophageal fistula, treated with stenting (A). Revealed fistulous opening 30 cm from incisor teeth (B). Revealed guidewire passes across the fistulous and position confirmed fluoroscopically and endoscopically (C). Revealed œsophageal stent (fully covered metal stent 12cm x 25cmm) placement done by passing it over guidewire. Proximal end of stent was kept at 21cm from incisor teeth and lower end kept above G-E junction.

**Figure 1:**
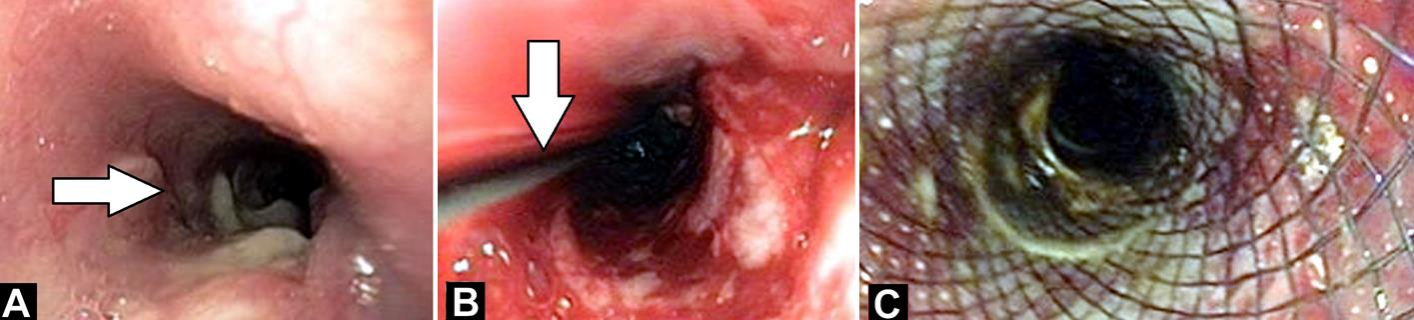
A) fistulous opening 30 cm from incisor teeth, B) guidewire passes across the fistulous and position confirmed fluoroscopically and endoscopically, C) œsophageal stent (fully covered metal stent 12cm x 25cmm) placement done by passing it over guidewire; proximal end of stent was kept at 21cm from incisor teeth and lower end kept above G-E junction

